# Synthesis of Ternary Borocarbonitrides by High Temperature Pyrolysis of Ethane 1,2-Diamineborane

**DOI:** 10.3390/ma8095285

**Published:** 2015-09-09

**Authors:** Fabrice Leardini, Lorenzo Massimi, Eduardo Flores-Cuevas, Jose Francisco Fernández, Jose Ramon Ares, Maria Grazia Betti, Carlo Mariani

**Affiliations:** 1 Departamento de Física de Materiales, Universidad Autónoma de Madrid, Madrid 28049, Spain; E-Mails: eduardoe.floresc@gmail.com (E.F.-C.); josefrancisco.fernandez@uam.es (J.F.F.); joser.ares@uam.es (J.R.A.); 2 Dipartimento di Fisica, Sapienza Università di Roma, Roma 00185, Italy; E-Mails: lorenzo.massimi@uniroma1.it (L.M.); maria.grazia.betti@roma1.infn.it (M.G.B.); carlo.mariani@uniroma1.it (C.M.)

**Keywords:** borocarbonitrides, graphitic materials, amine-borane adducts, thermolysis, X-ray photoelectron spectroscopy (XPS), Raman

## Abstract

Ethane 1,2-diamineborane (EDAB) is an alkyl-containing amine-borane adduct with improved hydrogen desorption properties as compared to ammonia borane. In this work, it is reported the high temperature thermolytic decomposition of EDAB. Thermolysis of EDAB has been investigated by concomitant thermogravimetry-differential thermal analysis-mass spectrometry experiments. EDAB shows up to four H_2_ desorption events below 1000 °C. Small fractions of CH_4_, C_2_H_4_ and CO/CO_2_ are also observed at moderate-high temperatures. The solid-state thermolysis product has been characterized by means of different structural and chemical methods, such as X-ray diffraction, Raman spectroscopy, Scanning electron microscopy, Elemental analysis, and X-ray photoelectron spectroscopy (XPS). The obtained results indicate the formation of a ternary borocarbonitride compound with a poorly-crystalline graphitic-like structure. By contrast, XPS measurements show that the surface is rich in carbon and nitrogen oxides, which is quite different to the bulk of the material.

## 1. Introduction

Solid hydrides have received a considerable attention in the last two decades within the framework of hydrogen storage applications. Among them, amine-borane adducts have been widely investigated in the last few years, due to their high hydrogen content, both in terms of gravimetric and volumetric capacities, and the moderate conditions needed for hydrogen desorption [1,2,3]. The prototypic and more-investigated amine-borane adduct is ammonia borane (NH_3_BH_3_, AB hereafter), which possesses the highest hydrogen gravimetric capacity among all amine-borane adducts and releases hydrogen at mild conditions. AB thermolysis takes place in three steps, evolving each step nearly one equivalent of H_2_ per AB equivalent. The first two steps occur at moderate temperatures (<200 °C) and give rise to the formation of polymeric amino borane ((NH_2_BH_2_)*_n_*) and polymeric iminoborane ((NHBH)*_n_*), respectively [4]. Small fractions of volatile by-products, such as diborane or borazine, are also observed. The third step occurs at much higher temperatures and gives rise to the formation of hexagonal boron nitride [5]. From the point of view of hydrogen storage applications, only the first two steps are of interest and have been investigated in detail. However, the use of AB as a precursor for the synthesis of hexagonal boron nitride (h-BN) has experienced an increasing interest among the scientific community in recent times [6]. Indeed, h-BN is a ceramic material, showing a high oxidation resistance at elevated temperatures, thus providing numerous applications. In addition, two-dimensional h-BN, also called white graphene, is a very interesting two-dimensional material due to its complementary properties as compared to graphene [7,8,9], as well as an ideal substrate to grow graphene [10]. The synthesis of white graphene is usually done by chemical vapor deposition, using AB as a precursor [11,12].

The previous examples show that the number of potential applications of solid hydrides, in particular of the amine-boranes, is well beyond the framework of hydrogen storage. In this work, we investigate the high temperature pyrolytic decomposition of a different amine-borane adduct, namely ethane 1,2-diamineborane (BH_3_NH_2_CH_2_CH_2_NH_2_BH_3_, EDAB hereafter). The synthesis of this compound was reported in the early 1960s through two paths, and its structural and vibrational properties were also investigated [13,14,15]. However, the thermolysis of EDAB has not been reported until recently. EDAB evolves between four to five equivalents of H_2_ per formula unit below 250 °C, forming a polymeric like derivative, with B–N and B=N bonds and maintaining CH_2_ groups in the chains [16,17]. A reasonably good reproducibility has been obtained comparing EDAB samples from different batches and synthetic routes.

It is expected that H atoms remaining in the polymer obtained at moderate temperature pyrolysis evolve at higher temperatures, thus forming a B*_x_*C*_y_*N*_z_* ceramic compound. It is therefore of interest to further investigate the pyrolysis of EDAB up to higher temperatures and characterize the obtained boro-carbo-nitrides. Hexagonal borocarbonitrides are of interest for the wide range of applications associated to the graphene-related technology, as well as for electrocatalysis and heat storage applications. Such compounds have been prepared following different synthetic approaches, usually using at least two precursors simultaneously [18]. This work investigates a novel synthetic approach towards the formation of ternary borocarbonitrides. It is a simple method using a single precursor, namely, the high temperature thermolysis of EDAB.

## 2. Results and Discussion

### 2.1. Structural Characterization of EDAB Precursor

The structural properties of the EDAB precursor have been investigated by X-ray powder diffraction (XRPD), as well as Fourier transformed infrared spectroscopy (FTIR). All diffraction peaks in the XRPD pattern of EDAB (see the Supplementary Information) can be indexed to *Pbca* space group characteristic of EDAB phase [19], with no traces of crystalline secondary phases. Rietveld refinement of the diffraction pattern gives the following lattice parameters, *a* = 10.709(1) Å, *b* = 8.134(1) Å and *c* = 8.092(1) Å, which are in good agreement with previously reported values [17,19]. Moreover, all modes appearing in FTIR spectrum of EDAB match quite well with previously reported [14,17], thus confirming the purity of the EDAB compound (see the Supplementary Information).

**Figure 1:**
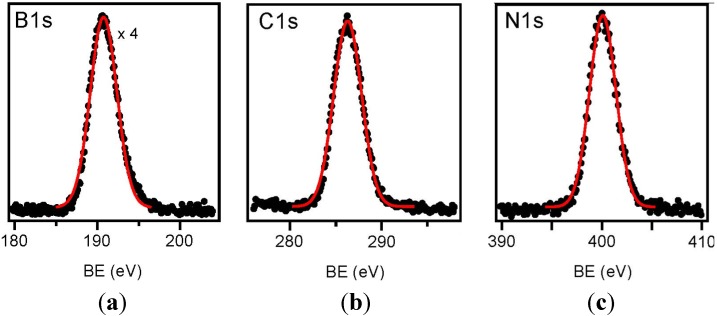
X-ray photoelectron spectroscopy (XPS) spectra of the (**a**) B 1s, (**b**) C 1s and (**c**) N 1s core levels of the pure molecule.

We also characterized the EDAB powder by X-ray photoelectron spectroscopy (XPS) prior to pyrolysis. As reported in Figure 1a–c B 1s, C 1s and N 1s core levels appear as single peaks localized at 191.0, 286.1 and 400.0 eV of binding energy (BE), respectively. The C 1s BE position is in good agreement to that typical of C atoms in sp^3^ hybridization, as expected in the pure molecule [20,21], and the B 1s and N 1s BE positions are in good agreement with those expected from boron, nitrogen and carbon mutually chemically coordinated with each other [22,23]. The B:C:N ratio estimated by the XPS signals, taking into account the atomic excitation cross sections [24], is 1:2.5:1, with a higher C content than expected in the pure molecule. This discrepancy can be explained by the presence of unavoidable carbon impurities at the very surface, since the molecular powder cannot be annealed (typical cleaning procedure to get rid of external impurities), in order to prevent its polymerization [16,17], and because XPS is a very surface sensitive technique. On the other hand, a bulk sensitive energy dispersive X-ray analysis (EDX) performed onto the powder before insertion into vacuum presents the correct ratio close to 1:1:1, thus confirming the purely surface nature of the excess carbon content.

### 2.2. High Temperature Thermolysis of EDAB Investigated by Combined Thermoanalytical Methods

**Figure 2:**
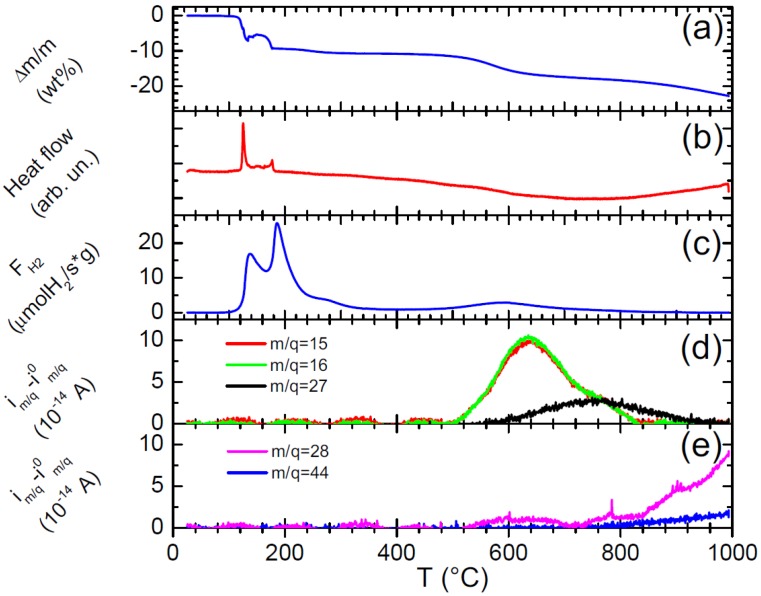
Differential thermal analysis-thermal gravimetric analysis-Mass spectrometry (DTA-TGA-MS) curves of ethane 1,2-diamineborane (EDAB) obtained under flowing Ar conditions at a heating rate of 2 K·min^−1^. (**a**) Relative mass loss (∆*m/m*) calculated from the TGA signal. (**b**) Heat flow recorded by the DTA apparatus. (**c**) Hydrogen desorbed flow (*F*_H2_) obtained from the i_2_ mass spectrometric signal. (**d**) Mass spectrometric ionic currents (*i_m/q_* − *i_m/q_*^0^, where *i_m/q_*^0^ are the corresponding baselines) at *m*/*q* = 15, 16 and 27 ascribed to CH_4_ and C_2_H_4_ desorption. (**e**) Mass spectrometric ionic currents at *m*/*q* = 28 and 44 ascribed to CO and CO_2_ desorption.

The thermolytic decomposition of EDAB at high temperature (up to 1000 °C) has been investigated by means of differential thermal analysis (DTA) coupled to thermal gravimetric analysis (TGA) and mass spectrometry (MS). The typical DTA-TGA-MS curve of EDAB recorded at 2 °C·min^−1^ under Ar flow is shown in Figure 2. It can be seen that EDAB starts decomposing at 100 °C, evolving pure H_2_ in a two-step exothermic process below 200 °C, as it was previously reported elsewhere [16,17]. Above that temperature, two additional H_2_ desorption events are observed, with peak maxima around 250 and 570 °C. These two processes seem to be almost thermoneutral in the DTA curve. A total amount of 6.8 equivalents of H_2_ evolved per EDAB equivalent has been obtained by integrating the MS signal. That value is slightly lower than the total theoretical amount of H_2_ in EDAB, namely, 7 equivalents. This result shows that some fraction of H remains in the sample, namely, about 0.2 H per EDAB equivalent, although almost all H is released by pyrolysis at 1000 °C. Whereas the first three H_2_ desorption steps observed below 400 °C have been ascribed to H atoms coming from N–H and B–H groups, the high temperature desorption event observed around 570 °C is related to H_2_ desorption from C–H groups in EDAB. In fact, the pyrolysis of C–H bonds usually takes place around that temperature, since C–H groups possess higher binding energies as compared to B–H and N–H ones. On the other hand, it can be observed that the mass loss recorded by TGA (−22.8 wt %) is slightly higher than the theoretical mass loss corresponding to H_2_ desorption (−15.6 wt %). The extra mass loss (7.2 wt %) must be ascribed to desorption of other molecules in addition to H_2_. In fact, MS measurements reveal additional desorption peaks at *m*/*q* = 14, 15, 16 and 27 around 570 °C, suggesting the release of CH_4_ and C_2_H_4_ molecules. Additional peaks are observed at *m*/*q* = 28 and 44 above 800 °C, indicating the release of CO and CO_2_. All other MS signals in the *m*/*q* = 3–100 range follow the same tendency than the baseline and their possible variations are within the error bars.

### 2.3. Characterization of the Thermolysis Product

**Figure 3:**
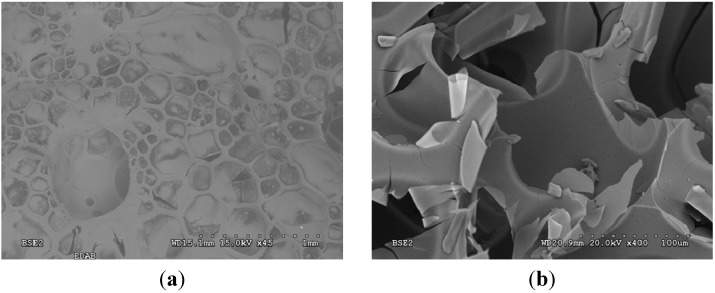
Scanning electron microscopy (SEM) micrographs in BSE mode (Back Scattered Electrons) of the obtained thermolysis products, taken at different magnifications: (**a**) 2220 × 2950 μm^2^ image size; (**b**) 240 × 320 μm^2^ image size.

The solid residue obtained after high temperature pyrolysis of EDAB has been characterized using different structural and chemical composition methods. Due to the fact that the thermolysis of EDAB is accompanied by significant sample foaming [17], the obtained residue appears in the shape of black foam. This sample foaming is also observed in the thermolysis of similar compounds, such as AB [1]. Typical scanning electron microscopy (SEM) images of the sample are shown in Figure 3, showing the cavities of the foamy-like products with a typical size in the 100–500 μm range.

Chemical composition has been characterized by means of EDX. These measurements give an approximate B:C:N ratio of 1:0.75:1. It must be noted that the B:C:N ratio of the EDAB precursor is equal to 1:1:1. The depleted amount of C as compared to B and N in the thermolysis product seems to be caused by the observed evolution of C*_n_*H*_m_* and CO*_x_* species during high temperature thermolysis of EDAB. A considerable amount of oxygen is also observed in EDX measurements, indicating the partial oxidation of the samples. Further characterizations were also done by means of Elemental analysis. This technique, however, could not provide reliable results since the samples presented a high resistance to oxidation even at 1000 °C and therefore, combustions were incomplete. This observation confirms the high resistance to oxidation exhibited by boron nitride and other borocarbonitrides. The C, N and H contents determined from the combusted fraction of the samples showed a C:N:H ratio of about 0.9:1:0.18. In spite of the limitations of Elemental analysis with these samples, it can be seen that C:N and N:H ratios agree reasonably well with those obtained from EDX measurements and from TGA-DTA-MS measurements, respectively.

**Figure 4:**
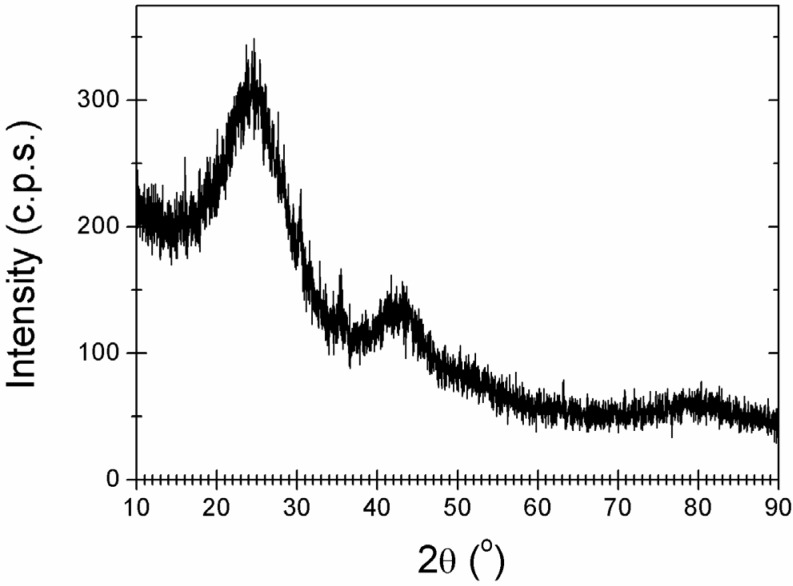
X-ray powder diffraction (XRPD) pattern of the product obtained by high temperature thermolysis of EDAB.

The structural properties of the obtained residue have been also investigated by X-ray powder diffraction, as shown in Figure 4. It is observed that samples present broad diffraction peaks centered at 2θ = 24.2° and 43.0°. It is worth to note that this diffraction pattern is similar to those reported for BN [5] and B*_x_*C*_y_*N*_z_* ternary compounds [25], which are isostructural to graphite. Accordingly, the diffraction peak at 24.2° has been related to (002) reflections, corresponding to the interlayer separation of the graphite-like domains. The peak at 43.0° has been related to the (100) reflections and corresponds to the in-plane lattice parameter of the hexagonal structure. In general, peak broadening in XRPD comes from the contribution of two terms: crystal domain size and lattice parameter variations. It must be noticed that hexagonal forms of carbon, boron nitride and borocarbonitrides are isostructural with a difference of less than 2% in their lattice parameters [7]. Such small differences in lattice parameter variations cannot account for the observed peak broadening. As a consequence, the observed peak broadening seems to be mainly due to the small size of crystalline domains, which is estimated to be of about 15 Å, according to Scherrer formula. The observation of a poorly crystalline phase upon thermolysis of AB at similar temperatures has been reported elsewhere [5].

**Figure 5:**
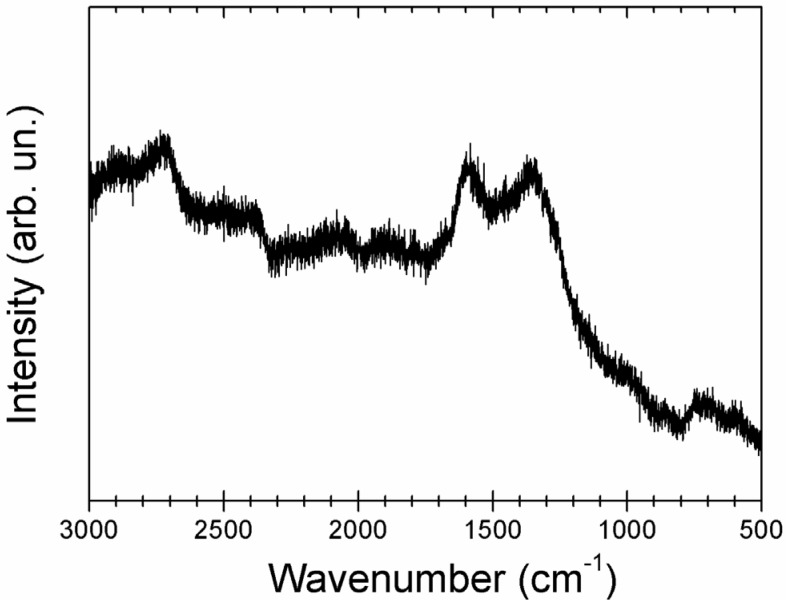
Raman spectrum of the sample obtained by high temperature thermolysis of EDAB.

The Raman spectrum of the EDAB thermolysis product is shown in Figure 5, the sample presents broad Raman modes centered at 1350, 1590 and 2715 cm^−1^. The Raman band at 1590 cm^−1^ has been ascribed to the G band observed in sp^2^ carbon systems and other borocarbonitrides [25]. Graphitic sp^2^ materials also exhibit a characteristic band (called 2D-band) around 2700 cm^−1^. Therefore, the occurrence of 1590 and 2715 cm^−1^ bands in the Raman spectrum shown in Figure 5 is a clear signature of the presence of graphitic carbon. As for the band appearing at 1350 cm^−1^, it has been assigned to in plane B–N vibrations, as usually observed in hexagonal boron nitride [5,18] and similar borocarbonitrides [25].

**Figure 6:**
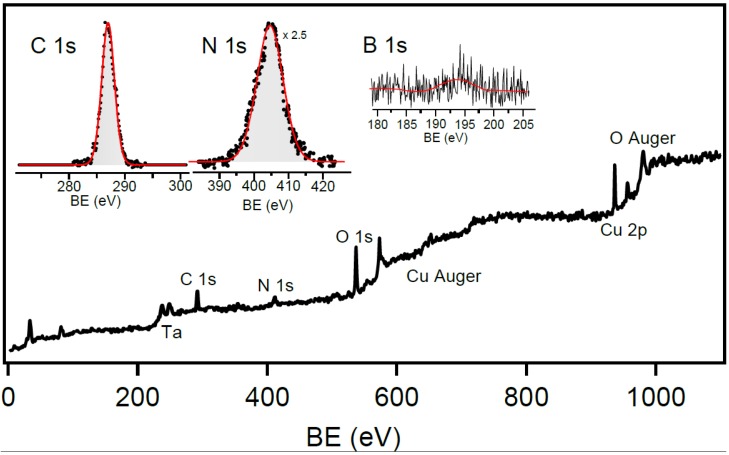
XPS spectra of the prepared sample after thermolysis, in ultra-high-vacuum (UHV). Complete spectral overview and detail of the C 1s, N 1s and B 1s core levels (insets).

In order to characterize the chemical state of C, N and B atoms in the obtained compound, we performed XPS measurements in ultra-high-vacuum (UHV) environment, *ex situ* after transferring the foamy sample into UHV. Before performing the XPS measurements, the sample has been annealed up to 300 °C in order to remove typical residual impurities adsorbed during the transfer. The XPS results are reported in Figure 6, the overview spectrum shows the presence of C, N and O as main peaks, negligible B, and Cu and Ta impurities due to the sample holder. In order to better characterize the core levels, the B, C and N 1s energy regions have been acquired with higher resolution: the C 1s core-level is characterized by a peak centered at about 287.0 eV of BE, in good agreement with previous literature about synthesis of hybrid B, C and N compounds [25], whose energy position is different from what is expected for a pure sp^2^ or sp^3^ hybridized carbon layer [21,22,25,26], it rather reflects the line shape and BE of C atoms bonded to oxygen [27,28,29]. The N 1s core level is characterized by a very broad peak localized at about 405 eV BE, which is not typical of N atoms bonded to carbon or boron [23,30]. This feature can be explained by coordination of nitrogen with oxygen atoms [31], and whose width (about 6 eV) suggests the presence of many different un-resolved chemical species. Finally, the B 1s core level appears as very small broad feature roughly centered at 192.8 eV, corresponding to the presence of tiny traces of boron oxide species [32,33]. Moreover, we highlight that our considerations are clearly supported by the presence of oxygen as observed in the wide range spectrum, despite the sample has been annealed to high temperatures in UHV to remove surface impurities. Oxygen present at sample surface has been ascribed to sample oxidation during the thermolysis of EDAB. In fact, by taking into account the purity of the Ar and H_2_ gases and the pressure conditions used in the thermolysis experiments, the H_2_O/O_2_ partial pressures are estimated to be in the 10^−3^ mbar range. By taking into account the length of these experiments, surface exposures to H_2_O and O_2_ are in the range 10^4^–10^5^ Langmuir. Due to the high temperatures used in the pyrolysis experiments, surface oxidation is expected to occur under these H_2_O and O_2_ exposures. Oxidized species are very stable and do not easily decompose by high temperature annealing (300 °C) under UHV, thus being the dominant species observed in the XPS spectra.

The B:C:N intensity ratio as estimated by XPS, showing a predominant amount of carbon, is different from what observed by the EDX analysis via SEM. This apparently contradictory result is due to the very surface sensitive character of XPS with respect to EDX. In particular, considering light elements such as carbon, the maximum estimated mean free path of the photoelectrons is less than 2 nm [34], much lower than the depth analyzed by EDX (about 100 nm), making XPS capable to extract information only from the very first atomic layers. From this observation, our results clearly demonstrate that the bulk of the synthesized compound explored by EDX/SEM, XRD and Raman is strongly different from the surface: while the bulk of the foamy material is characterized by formation of the borocarbonitride compounds, the surface carbon and nitrogen species are predominant with respect to boron, and appear as strongly oxidized, as also confirmed by the presence of oxygen in the XPS spectra. However, we are not able to provide an accurate B:C:N ratio, since XPS performed with this photon energy is not the best technique to quantify light elements in low concentrations. In fact, in the experiment performed with 1486.7 eV photon energy, the light elements like B present a very low excitation cross-section [24,35]. Due to these limitations, we cannot quantify the B content, but we can infer general considerations about the composition, in particular at the surface.

This combined bulk/surface sensitive spectroscopy analysis highlights how the *ex situ* synthesized borocarbonitride is a bulk material, whose surface is prevalently composed by C and N oxidized species covering the compound. These complementary measurements, thus, strongly suggest to perform *in situ* in-vacuum synthesis and characterization from EDAB to borocarboniride, a fundamental step in order to obtain and investigate the EDAB pyrolysis avoiding oxygen contamination, thus reaching the formation of a pure hybrid BCN phase, even at the very surface.

## 3. Experimental Section

Ethane 1,2-diamineborane (EDAB) has been supplied by Boron Specialties and used without further purification. Thermolysis of EDAB has been investigated by concomitant DTA-TGA-MS measurements (under an Ar flow of 40 sccm) in a DTA-TGA system (Setaram Setsys Evolution 1200, Setaram, France) coupled to a quadrupole mass spectrometer (QMS, Pfeiffer, Switzerland). The H_2_ detection sensitivity of QMS has been calibrated at the same experimental conditions, as described elsewhere [17]. Additional thermolysis experiments were performed in a quartz tube inserted in a tube furnace (Nabertherm Controller B 170, Nabertherm, Germany) with larger amounts of EDAB, in order to produce samples for XRPD, XPS and other characterization techniques. In those synthesis experiments temperature was monitored by using a R-type thermocouple placed outside the quartz tube. These thermolysis were done while heating under 85% Ar-15% H_2_ flow at a heating rate of 16 °C/min up to 1000 °C. The purity of the Ar and H_2_ gases was 99.999%. The total pressure during the thermolysis experiments was always the atmospheric pressure and no pressure built-up occurred due to the gas evolution observed during the pyrolysis. The quartz tube and gas lines were checked for leaks by using an Inficon Sensitor ISH2000 hydrogen detector. EDAB was dissolved in anhydrous tetrahydrofuran (THF, Sigma Aldrich) and then deposited onto a Cu foil (Alfa Aesar, 25 μm), which served as an inert substrate [7,8,9] to growth the ternary borocarbonitrides.

X-ray diffraction measurements have been done in a X’Pert PRO diffractometer (Panalytical) with θ/2θ geometry by using Cu K-α radiation. A zero diffraction plate for XRD sample holder has been used (silicon single crystal cut at special orientation). FTIR measurements have been done in transmission mode in a Bruker IFS66v apparatus, by mixing EDAB with KBr (1 wt % of EDAB) and pressing the powders into circular pellets. Scanning electron microscopy has been done in a Philips XL30 apparatus equipped with energy dispersive X-ray analysis (EDAX Dx4i). Elemental analysis has been performed with a LECO CHNS-932 apparatus. Raman spectra have been acquired in a Labram HR Raman spectrometer (Horiba Scientific, Japan) by exciting with a 532 nm laser.

X-ray photoelectron spectroscopy (XPS) measurements of the B 1s, C 1s and N 1s core-levels were carried out at the LoTUS surface physics laboratory (Sapienza, University of Rome) in an Ultra High Vacuum (UHV) chamber, with a base pressure in the mid 10^−10^ mbar range. XPS spectra were acquired with Al K-α radiation (hν = 1486.7 eV), and electrons measured with the hemispherical analyser VG Microtech Clam-2 (VG Microtech, Uckfield, UK), in constant pass energy (PE) mode set at 100 eV. The binding energy (BE) was calibrated by acquiring after each measurement the Au 4f_7/2_ core-level at a BE = 84.0 eV.

## 4. Conclusions

The present work reports a novel synthesis route towards the formation of ternary borocarbonitrides. It is based on the use of a single precursor containing B, C and N. Such precursor is ethane 1,2-diamineborane (EDAB), a linear molecule of BH_3_, NH_2_ and CH_2_ groups, chemically isoelectronic to hexane. The high temperature thermolysis of EDAB has been investigated by TGA-DTA-MS experiments. EDAB shows up to four H_2_ desorption events below 1000 °C. Small fractions of CH_4_, C_2_H_4_ and CO/CO_2_ are also observed at moderate-high temperatures. This leads to the formation of a B*_x_*C*_y_*N*_z_* compound with traces of oxygen and some residual H. The obtained samples have been characterized through chemical and structural methods such as SEM, EDX, Raman and XPS, confirming the formation of a poorly crystalline borocarbonitride compound. The XPS measurements, revealing a highly oxidized surface layer with respect to the bulk compound, suggest further investigation aiming at the EDAB pyrolysis in controlled UHV conditions.

## Supplementary Materials

Supplementary materials can be accessed at: http://www.mdpi.com/1996-1944/8/9/5974/s1.
