# Supplementary material for: Synthesis of Ternary Borocarbonitrides by High Temperature Pyrolysis of Ethane 1,2-Diamineborane
Source: Materials (Basel). 2015 Sep 9;8(9):5974–85. doi: 10.3390/ma8095285 (PMC5512665; doi:10.3390/ma8095285)
Supplement: Supplementary file 1 [file materials-08-05285-s001.pdf]

Supplementary Materials

EDAB sample supplied by Boron Specialties has been characterized by means of X ray powder diffraction (XRPD) and Fourier Transform Infrared Spectroscopy (FTIR).

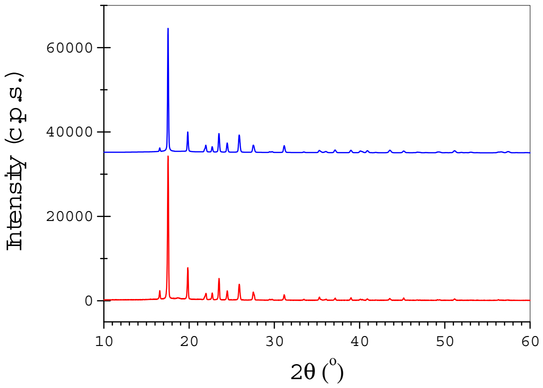

Figure S1. X-ray Powder Diffraction Pattern of ethane 1,2-diamineborane (EDAB). Upper curve (in blue) represents experimental data points whereas the bottom line (in red) is the simulated profile by considering an orthorhombic cell of *Pbca* space group corresponding to EDAB phase [1–3]. Experimental curve has been vertically shifted to allow a better comparison with simulated diffraction pattern.

Peak positions of the main IR absorption bands of EDAB are indicated in Table S1. Values previously reported for EDAB, as well as the assignment of each band, are also included for comparison purposes.

Table S1. Peak positions (in cm^{-1}) of the main IR absorption bands of EDAB (vs: very strong; s: strong; m: medium; sh: shoulder). Tentative assignments of each band are also indicated (str.: stretching mode; sym.: symmetric; asym.: asymmetric; ds: deformation scissors; d.: deformation).

Ref. [4]	Ref. [3]	This work	Assignment	Ref. [4]	Ref. [3]	This work	Assignment
3240	3262s	3274s, 3249s	N–H asym. str.	1359	1360s	1361s	-
3200	3225s	3229s, 3222s	N–H asym. str.	1262	1262s	1261s	C–H d
3120	3145m	3145s	N–H sym. str.	1257	1256s	1256sh	N–H d
2970	2992m	2992s	C–H asym. str.	-	1202sh	1200sh	N–H twist
2955	2965m	2965s	C–H asym. str.	1189	1189s	1188vs	B–H d
2880	2896m	2896m	C–H sym. str.	1172	1172s	1173vs	B–H d
-	2850s	2846m	C–H sym. str.	1165	1163s	1162vs	B–H d
2370	2397s	2393s	B–H str.	1125	1125sh	1124m	B–H d
-	2345s	2337vs	B–H str.	1041	1042s	1042s	C–N str.
2315	2326s	-	B–H str.	1008	1010s	1010s	-
2275	2271s	2273vs	B–H str.	929	928s	930s	-
-	1591s, 1586s	1591sh	N–H ds	783	784s	784s	-
1583	1581s	1581s	N–H ds	760	762m	763m	-
1467	1473m, 1466s	1467s	C–H ds	705	706s	707s	^{11}B–N str.
1366	1368s	1365sh	N–H d	-	-	-	-

References

1. Ting, H.Y.; Watson, W.H.; Kelly, H.C. The Molecular and Crystal Structure of Ethylenediamine-Bisborane, $C_2H_{14}B_2N_2$. *Inorg. Chem.* **1972**, *11*, 374–377.
2. Neiner, D.; Karkamkar, A.; Bowden, M.; Choi, Y.J.; Luedtke, A.; Holladay, J.; Fisher, A.; Szymczak, N.; Autrey, T. Kinetic and thermodynamic investigation of hydrogen release from ethane 1,2-di-amineborane. *Energy Environ. Sci.* **2011**, *4*, 4187–4193.
3. Leardini, F.; Valero-Pedraza, M.J.; Perez-Mayoral, E.; Cantelli, R.; Bañares, M.A. Thermolytic decomposition of ethane 1,2-diamineborane investigated by thermoanalytical methods and *in situ* vibrational spectroscopy. *J. Chem. Phys. C* **2014**, *118*, 17221–17230.
4. Goubeau, J.; Schneider, H. Borin-Anlagerungsverbindungen des Äthylendiamins. *Chem. Ber.* **1961**, *94*, 816–821.
